# Public health round-up

**DOI:** 10.2471/BLT.14.010914

**Published:** 2014-09-01

**WHO calls for an end to attacks on Gaza health facilities**

The in-patient department of Alaqsa hospital south of Gaza City after it was attacked by air missiles on 21 July. According to the Ministry of Health in Gaza City, 15 of 32 hospitals have been damaged since violence erupted in June and 10 of them (including six of the damaged ones) have been closed. Three of the incidents – including the one on 21 July – caused dozens of casualties among staff and patients. Since June, five ambulance workers have been killed and 40 injured in the conflict. The World Health Organization has called for a halt in the attacks on health-care facilities, staff and vehicles in the Gaza Strip.

**Figure:**
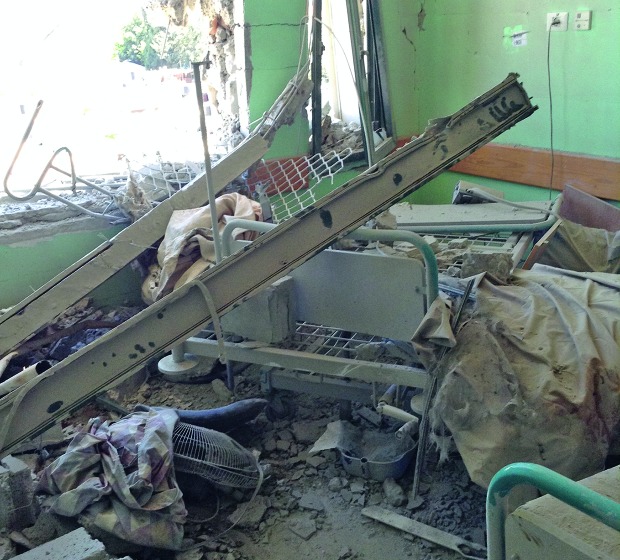
WHO/Mahmoud Daher

## WHO coordinates Ebola outbreak response

WHO Director-General Dr Margaret Chan has taken charge of coordinating the international response to the Ebola virus disease outbreak in west Africa to prevent its spread beyond the four countries that have already been affected.

The Director-General launched the Ebola Virus Disease Outbreak Response Plan in West Africa at a meeting with the presidents of Guinea, Liberia and Sierra Leone on 31 July. The plan envisages raising US$ 100 million in funds for the task at hand. 

“The scale of the Ebola outbreak, and the persistent threat it poses, requires WHO and Guinea, Liberia and Sierra Leone to take the response to a new level, and this will require increased resources, in-country medical expertise, regional preparedness and coordination,” the Director-General said. “The countries have identified what they need, and WHO is reaching out to the international community to drive the response plan forward.”

On 8 August, WHO declared that the international spread of Ebola virus disease constitutes a Public Health Emergency of International Concern or PHEIC, following advice from a committee of international experts. The decision came days after confirmation that the outbreak had spread beyond Guinea (where it is believed to have started in December 2013), Liberia and Sierra Leone to a fourth country, Nigeria. 

The committee of experts warned that the consequences of further international spread of the disease would be particularly serious given the intensive community and health facility transmission patterns, and the weak health systems in the region. 

A coordinated international response is essential to stop the disease spreading to other countries, they said.

The outbreak response plan calls for hundreds more staff – especially physicians and nurses, epidemiologists, social mobilization experts, logisticians and data managers – to be deployed in the affected countries, where treatment facilities are badly overstretched. 

Mobilizing communities and boosting communications is a vital part of the plan, so that people know how to avoid infection and what to do if they fear they have been infected. Improving the prevention, detection and reporting of suspected cases and referring people who have become infected with the virus for medical care, as well as psychosocial support, are also key to the plan. 

“The situation in West Africa is of international concern and must receive urgent priority for decisive action at national and international levels. Experiences in Africa over nearly four decades tell us clearly that when well managed, an Ebola outbreak can be stopped,” Chan said.

In July, WHO opened a Sub-regional Outbreak Coordination Centre in Conakry. Hundreds of international aid workers and nearly 150 WHO staff were already supporting the response in the centre by the second week of August. 

The scale of the Ebola virus disease outbreak is unprecedented, with 2473 cases reported and 1350 deaths in Guinea, Liberia, Nigeria and Sierra Leone (reported 22 March to 18 August 2014).

http://www.who.int/csr/disease/ebola/en/

## New WHO report on suicide prevention

The scientific literature on suicide prevention has grown in recent years but the taboo and stigma surrounding suicide persists, and many people at risk fail to seek help while health systems often fail to provide timely, effective support, according to a new WHO report.

The report, which will be released on 5 September, proposes a systematic evidence-based approach that countries can take to develop suicide prevention policies and programmes. It is the first global report that WHO has devoted to the subject and it identifies the key factors that lead to suicide. 

“Policies aimed at reducing access to the means of suicide, such as access to pesticides or firearms, as well as to promote responsible media reporting of suicide, and to reduce the harmful use of alcohol are key public health interventions to preventing suicide,” said Dr Shekhar Saxena, director of the Department of Mental Health and Substance Abuse at WHO. 

Early identification and treatment of depression and alcohol use disorders are also key for the prevention of suicide, as well as follow-up contact with those who have attempted suicide. 

The report calls for a public health approach involving many sectors to tackle the problem including education, employment, agriculture, social welfare, the judiciary and others. It is being released ahead of World Suicide Prevention Day on 10 September. 

http://www.who.int/mental_health/suicide-prevention/en (from 5 September)

**Cover photo**

This photograph of a Moroccan Berber carrying a child on her back is part of a series called Birth Day, by Belgian photographer Lieve Blancquaert, that tells 14 stories about childbirth from different cultures around the world. This and other photographs from the series were in an exhibition at WHO headquarters in Geneva this year.

**Figure:**
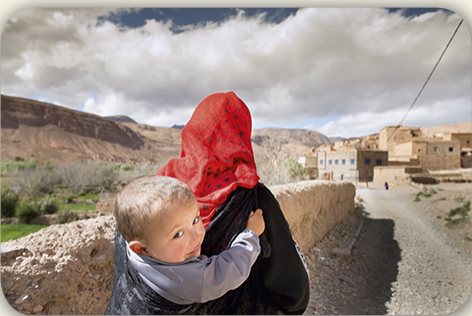
Courtesy of Lieve Blancquaert

## Medical assistance reaches Syrians in need 

The World Health Organization and the Syrian Arab Red Crescent (SARC) crossed enemy lines to deliver surgical and medical assistance to besieged areas of eastern Aleppo city and around the town of Mouadamiya, thanks to a ceasefire agreement with the Syrian government and the opposition in July. 

Five tonnes of medical equipment were delivered to Mouadamiya to support a population of 24 000 people on 28 July. In addition, antibiotics, multivitamins for children and two months’ supply of medicines for chronic diseases were delivered to a health centre in Mouadamiya. 

Two days later, 10 tonnes of surgical supplies – including more antibiotics, sterile gauze, syringes and catheters – as well as medicines for chronic diseases for 22 000 people were delivered to four hospitals in eastern Aleppo city.

“These unprecedented deliveries will bring much needed surgical supplies and medical treatments to vulnerable populations in two of the most hard-to-reach and deprived areas in the Syrian Arab Republic,” said Elizabeth Hoff, WHO Representative in the country. She added that WHO is working with partners and the Syrian health authorities to turn these deliveries into a long-term supply for both areas.

The humanitarian situation has been dire with both areas reporting severe shortages of basic medicines and life-saving supplies since the conflict started in 2011. The deliveries were authorized and eased by United Nations Security Council Resolution 2165 (2014) which aims to get more aid delivered to 10.8 million Syrians in need through the most direct routes. 

WHO Syria is working closely with the Syrian health authorities to facilitate the implementation of this resolution.

Since the beginning of 2014, WHO has delivered life-saving medical assistance to over 7 million Syrians in need across the country, including in besieged, hard-to-reach and opposition-controlled areas.

http://www.emro.who.int/media/news/who-sarc-reach-aleppo.html

## Putting people first

The science and practice of people-centred health systems will be the theme of the next Global Symposium on Health Systems Research, a five-day meeting that starts this month in Cape Town, South Africa. 

“People-centred health systems is a relatively new term and there is debate about its meaning,” said Dr Lucy Gilson, co-chair of the programme working group and head of the Symposium Secretariat. 

“There is growing recognition that to achieve universal health coverage, the quality of the health care is as important as the ability to provide affordable services that are accessible – and that´s where people-centred health systems are vital,” Gilson said. 

Thousands of participants are expected to attend the international meeting, which is co-sponsored by the Alliance for Health Policy and Systems Research, a WHO partnership. 

Health Systems Global, a membership organization that was launched at the last Symposium in Beijing, is another co-sponsor along with the local organizing consortium of leading South African research institutions. The Symposium from 30 September to 3 October is also being supported by the Department of Health of South Africa.

http://hsr2014.healthsystemsresearch.org/

**Looking ahead**

**6–8 October – WHO expert consultation on ageing and health** at WHO headquarters to follow the international day of older persons on 1 October
**10 October – World Mental Health Day** with the theme of living a healthy life with schizophrenia. http://www.who.int/mental_health/world-mental-health-day/2014/en/
**12 November – World Pneumonia Day**
**14 November – World Diabetes Day**
**19–21 November 2014 – Food and Agriculture Organization/WHO Second International Conference on Nutrition (ICN2)** at FAO headquarters, Rome, Italy. http://www.who.int/mediacentre/events/meetings/2014/international-conference-nutrition/en/
**1 December – World AIDS Day**
**3–6 December – World Cancer Congress** in Melbourne, Australia. http://www.worldcancercongress.org/

